# Supplementary material for: Are carbon emissions trading and green financial instruments synergistic? -Comprehensive quantitative research based on content analysis
Source: PLoS One. 2024 Mar 7;19(3):e0298601. doi: 10.1371/journal.pone.0298601 (PMC10919601; doi:10.1371/journal.pone.0298601)
Supplement: S1 Data — (ZIP) [file pone.0298601.s001.zip › Data/地级市Z_F工作报告.zip!/╣π╢1⁄2/╔╪╣╪╩╨/└┤╫╘╣1⁄2╓┌║┼í╛┬φ┐╦╩2╛▌═°í┐_2010.docx]

各位代表：

我代表市人民政府，向大会作政府工作报告，请予审议，并请市政协委员和列席人员提出意见。

2009年工作回顾

2009年是我市有效应对国际金融危机、各项工作取得明显成绩的一年。在党中央、国务院和省委、省政府及市委的正确领导下，在市人大及其常委会、市政协的监督支持下，市政府团结带领全市人民，深入学习实践科学发展观，积极落实省委、省政府“三促进一保持”和“双转移”工作部署，抢抓实施珠江三角洲地区改革发展规划纲要带来的机遇，攻坚克难，顽强拼搏，实现了市十二届人大四次会议确定的主要发展目标，经济社会发展继续呈现良好的上升势头。

一、经济保持平稳较快增长，发展质量逐步提高

据统计，2009年全市实现生产总值571.7亿元，比上年增长10.1%（按可比价格计算），其中第一产业增加值增长6.6%，第二产业增长8.1%，第三产业增长13.0%。人均生产总值1.9万元，增长9.8%。三次产业结构得到优化。

加强对工业的扶持、协调和服务，有效应对新世纪以来最为困难的工业形势，工业增加值扭转了连续三个季度下降的局面，全年增长6.1%。七大支柱工业中，制药和玩具工业规模迅速扩大，烟草和有色金属工业较快增长，钢铁、机械和电力工业稳步回升，困难企业普遍走出困境。钢铁产业链条延长，钢铁、钢材产量实现增长。韶钢节能减排技改项目进展迅速。丹霞冶炼厂改扩建一期工程竣工投产。

科技创新和成果转化取得新突破。利民制药厂“现代中药参芪扶正注射液的研究与产业化”项目和坪石发电B厂参与的“湿法烟气同时脱硫脱硝成套技术研究与应用”项目通过2009年度广东省科学技术奖一等奖评审。企业研发成果实现转化。韶钢集团成为我省“百强创新型企业培育工程示范企业”。深化产学研合作，液压件行业与全国知名高校、科研院所联合建立液压机械装备产学研创新联盟。我市分别与中国科学院、华南理工大学达成重要合作意向并逐步落实。实施一批省级以上重大科技专项，新增一批国家级高新技术企业、省级民营科技企业、创新型企业、工程技术研发中心和高新技术产品。乳源铝箔产业基地成为我市首个省级产业集群升级示范区。全市专利申请量514件，居全省山区市首位。全市有省著名商标16件，新增4件。东阳光公司的“HEC”商标成为我市首个“中国驰名商标”。我市首次通过全国科技进步考核（2007—2008年度）。市中小企业创业基地成为省中小企业创业示范基地。全市个体工商户81388户、资金数额34.7亿元，分别增长7.5%和7.7%；私营企业6975户、注册资本127.2亿元，分别增长10.2%和7.2%。民营工业发展加快，全市民营经济增加值增长12.0%。

城乡市场繁荣，服务业持续较快发展。市场经济秩序进一步规范。居民价格指数涨幅得到有效控制。全市社会消费品零售总额287.7亿元、增长19.2%。商品房销售面积和销售额分别增长62.6%和82.1%。全市接待旅游者人数1226.1万人次、增长17.4%，旅游总收入71.4亿元、增长35.7%。整合跨行业资源，初步形成旅游信息服务网络，建成国内领先的旅游信息服务平台，与省信息产业厅、省旅游局共建的我省首个旅游信息化示范区顺利推进。丹霞山被评为全国十大文化生态景区，并与美国泽恩国家公园缔结为国际姊妹公园。广东大峡谷成为国家 4A 级旅游景区。开通运行“韶关物流信息网”，启动曲江、龙归、浈江等综合物流园和韶关市口岸通关物流中心的规划建设。韶钢港务、韶峰等物流企业发展壮大。

财政运行良好，金融发展提速。全市地方财政一般预算收入40.7亿元，增长11.9%。农业农村和教育卫生支出大幅增加。组建政府全资的政策性担保公司，发展两家小额贷款公司，拓宽了企业、个体工商户和“三农”融资渠道。年末金融机构本外币存款余额794.4亿元、贷款余额320亿元，比年初分别增长17.8%和44.6%；存贷比为40.3%，比上年提高7.5个百分点。保险业金融机构保费收入16.9亿元、增长12.1%。证券业金融机构交易额达1395.4亿元，增长89.0%。金融对全市经济增长的贡献明显提升。

城乡居民收入持续增长。城乡居民人均收入8893元、实际增长11.6%，其中城镇居民人均可支配收入12948元、实际增长10.8%，农村居民人均纯收入5338元、实际增长12.9%。在岗职工年人均工资28000元，增长10.8%。年末城乡居民储蓄存款余额489亿元，增长12.8%。

县域经济发展亮点纷呈。各县（市）生产总值、财政收入增速均超过全市平均水平。南雄精细化工、乳源氯碱化工、仁化有色金属循环经济、始兴制笔研发制造等特色专业园区建设取得明显成效。乳源被评为“中国果菜无公害十强县”。南雄荣登“中国信息化最受关注城市榜”。始兴被评为自然生态“中国最美的小城”。乐昌成为广东旅游强县（市）。粤台农业合作示范区落户翁源。

二、投资规模不断扩大，发展后劲进一步增强

全社会固定资产投资完成356.5亿元，增长25.6%。重点项目完成投资192.9亿元，为年度计划的122.9%。韶关电网建设与改造、坪石发电B厂三期、韶关碧桂园、风度国际大酒店等项目超额完成全年投资计划。乐昌峡水利枢纽成功截流，湾头水利枢纽完成主体土建工程。乳源赛普超硬材料等8个重点预备项目开工建设。韶关核电项目前期工作扎实推进。全年争取中央和省扩大内需项目专项资金19.6亿元。

重大交通基础设施项目如期推进。武广高速铁路韶关段、韶关站场建成运营，我市融入广州“1小时生活圈”，区位交通优势进一步提升。韶赣高速公路南雄段春节前可建成通车。赣韶铁路、广乐高速公路开工建设。粤湘、大广、昆汕高速公路韶关段前期工作继续推进。韶关至贺州铁路列入部省共建项目。完成国、省道改造 30.6公里 、路面大中修 105公里 。

三、农业农村稳步发展，强农惠农政策全面落实

克服去年秋季严重干旱影响，农林牧渔业普遍增产增收。全市农业增加值增长6.6%，增幅创多年来新高。农作物播种面积增加5.2%，粮食大面积丰收、总产量增加6.6%，完成粮食考评目标任务。完成粮食清仓查库工作，全市粮食库存账实相符、管理规范。生猪饲养量增长11.0%，出栏量增长12.1%。肉类总产量增长10.2%。粤北现代农业示范园区建设扎实推进。农业产业化、标准化、信息化步伐加快。市级农业龙头企业销售收入增长11.1%，带动15.3万农户，户均增收3968元。农民专业合作社达256个，新增115个。农业招商引资势头上升，引进项目22宗，总投资11.9亿元。获无公害农产品、绿色食品、有机农产品认证的企业（单位）累计达74家，新增38家。

全市落实各类农业补贴1.8亿元。统一全市商品木材规费项目和征收标准，减轻了林农林企负担。全面部署实施扶贫开发“规划到户、责任到人”工作。加强耕地保护和土地执法监察。完成耕地开垦8.2万亩。改造中低产田3.8万亩，改良土壤1.8万亩。农村公路硬化完成 610公里 ，改造县道 83公里 。农民改造农房1.2万户、安装太阳能热水器5000户、新建户用沼气池2500个。完成水库移民建房530户。建成农家书屋83家、农民健身工程586个。推进乡村“清洁美”和“万村绿”工程，开展村庄规划整治，农村面貌不断改观。

四、“双转移”步伐加快，掀起“两个高潮”初见成效

着力掀起园区基础设施建设、招商引资两个高潮。承接产业转移取得新成绩。举办大型推介招商活动和参加国际国内经贸活动签约项目投资总额超过1000亿元。莞韶产业园签订合同项目数增长5倍，投资总额增长6倍，实际到位资金增长76.2%。深圳比亚迪公司在我市兴建国内最大的试车场和汽车零部件项目开工建设。韶关、广州两市政府签署加强汽车及零部件产业合作框架协议。宏大齿轮、正星车轮配套广汽日野取得实质性进展。

园区开发建设加快。全市产业转移工业园完成固定资产投资22.4亿元，增长54.1%；建成规模以上工业企业72户。莞韶产业园完成基础设施建设投资6.9亿元、增长1.2倍，工业增加值增长20.3%；与银行签订贷款批准额度12.3亿元，引导社会资金投入13.5亿元，完成征地1.2万亩。

农村劳动力技能培训转移就业工作加强。集劳动力市场、人才市场、大中专毕业生就业服务于一体的市人力资源市场建成启用。农村劳动力转移技能培训2.8万人，转移就业9.3万人，分别增长55.0%和40.6%，均超额完成省下达任务。经培训鉴定合格后的农村劳动力就业率达85%以上。

外经贸工作取得新成绩。全年实际吸收外资1.9亿美元，增长4.0%。外贸出口5.8亿美元，比上年略有增长。引进世界500强企业和企业“走出去”均取得新突破，日本三井物产株式会社与我市企业合资经营汽车铝箔，凡口铅锌矿收购澳大利亚PEM公司50.1%股权。韶关铁路口岸铁海联运项目成功竞得省口岸建设补助资金1000万元。口岸通关货运量450万吨，增长10.0%。铁海联运启用顺畅。我市与深圳盐田港签订了共建韶关口岸通关物流中心框架协议。

五、城市建设取得新成绩，生态文明建设有新进展

芙蓉新城征地工作基本完成，下胡村安置区动工建设。韶关大道全线通车。芙蓉大道南段建设启动。芙蓉山国家矿山公园、韶阳楼和韶关城市规划展示厅建成开放。北伐纪念馆完成主馆建设。宝盖路建成通车。林桥坑一期整治完工。市区新敷设供水主管 17公里 ，更新公交车86辆，开工建设防洪堤 18.2公里 。

全市完成造林作业面积57.2万亩、增长40.2%，森林覆盖率由2008年雨雪冰冻灾害后的64.6%上升到66.8%，森林生态恢复加快。加强地质灾害防治和矿山资源整治。完成水土流失治理面积9平方公里。预计全年万元工业增加值能耗下降4%，完成省下达的节能减排任务。翁源、乳源、仁化、乐昌坪石污水处理厂和曲江污水处理厂二期竣工，完成省下达的“一县一厂”建设任务。市二污厂二期春节前可通水试运行，市区生活污水集中处理率提高到80%以上。

六、社会文明进步，各项事业不断发展

以建设社会主义核心价值体系为根本，深入推进思想道德建设和精神文明创建活动。开展以“身边好人·感动你我”为主题推荐评选活动，树立、宣传了一批道德典型，参与中央文明办主办的“我推荐、我评议身边好人”活动，邓玉莲、林锐生等18人荣登“中国好人榜”。中央文明办在我市成功举办全国道德模范与身边好人现场交流活动。广泛开展文明礼仪教育实践活动，组织开展“志愿者服务月”、红十字志愿服务、绿网志愿者等活动。涌现大批精神文明建设先进集体和先进个人。抓好整治互联网低俗之风、集中整治网吧、净化荧屏声频和治理校园周边环境四大专项行动，营造良好的社会环境，促进未成年人健康成长。认真落实优抚安置政策，深入开展军民共建和拥军优属活动，加强国防教育和民兵预备役工作，建立健全抚恤补助标准自然增长机制，建立优抚医疗保障制度。安全生产形势持续向好，事故起数、伤亡人数和直接经济损失均继续下降。加强人民调解工作，化解社会矛盾纠纷。“六好”平安和谐社区建设稳步推进，完成27个“六好”平安和谐社区创建工作，武江区新华街道惠民西社区成为全国平安和谐社区示范单位。全市建立“无传销社区”508个，通过省政府打传工作考核。及时、妥善处置旭日玩具厂“6·26”斗殴事件。加强社会治安综合治理，开展多个专项行动，严厉打击突出违法犯罪，维护了社会稳定。

创建省教育强市工作扎实推进。办学条件进一步改善，全市新增校园面积19. 3 万平方米 、校舍17. 3 万平方米 。规范化学校建设成效显著，义务教育发展水平进一步提升。高中阶段教育发展加快，办学规模继续扩大，毛入学率提高到88%。中等职业技术教育招生5.8万人，增长36.1%。韶关学院、松山职业技术学院、市电大等三所高校招生超过1万人，增长18.6%。学前教育、特殊教育和民办教育稳步发展。学校管理和教师队伍建设得到加强，教育质量和办学水平进一步提高。

文化惠民工程深入实施，公共文化服务体系进一步完善。抗战时期中共广东省委暨粤北省委机关旧址得到保护维修。5个项目入选省级非物质文化遗产名录。群众文化活动蓬勃开展。文艺成果获省第七届“五个一工程”奖1项、第九届“百歌颂中华”歌咏大赛金奖2项、第六届群众戏剧曲艺花会奖2金2银。颁发首届文艺创作“风度奖”。基层文化信息资源共享工程和流动综合文化服务工程取得新进展。

医疗服务区域中心建设加快，重点学科、特色专科建设取得重大进展，市区医院对周边地区的服务辐射明显。公共卫生服务能力加强，甲型H1N1流感等重大疾病得到有效控制。城乡卫生服务体系建设加强。新型农村合作医疗筹资和保障水平提高，参合率达98.8%。加强食品药品安全专项整治，反映药品生产和流通领域质量总体水平和状态的评价性抽检合格率100%，为历年最高。完成省下达的人口计生工作任务，低生育水平持续稳定。成功举办韶关市第十三届运动会、第四届社区体育运动会和第三届老年人运动会。民族宗教、外事侨务、统计、对台、港澳、气象、水文、防震减灾、无线电管理、供销、档案、人防、史志、残疾人、慈善、红十字会、老龄、妇女儿童等各项工作取得新成绩。

七、改善民生力度加大，困难群众基本生活得到保障

城镇新增就业5.3万人、增长2.4%，城镇失业人员再就业4.2万人、增长2.1%，城镇登记失业率为3.2%。大中专毕业生就业率79.2%，比上年提高4.2个百分点。全市养老、医疗、失业、工伤、生育基金征集收入增长20%。城镇居民基本医疗保险缴费人数增长15.2%。开展了新型农村社会养老保险试点工作。全市有11.1万人享受低保待遇，支出低保资金1.1亿元。发放医疗救助金救助了1.1万人。韶关、坪石两个救助站合计救助5395人（次），救治流浪乞讨精神病患者35名。2770名农村贫困家庭子女免费接受中等职业技术教育。办理法律援助案件1081件，增长36.1%。保障性住房建设完成年度目标任务，建成廉租住房和经济适用住房1266套。启动市区人才住房解困工作，建成第一批人才公寓27套。

去年市政府承办的十件实事中，农村劳动力转移就业培训1.5万人、19万农村人口食水安全工程、市人力资源市场、村庄整治110个、建设全市旅游公共标识系统、市区更新增添一批公交车辆等6件实事均已完成，帽峰大桥超额完成年度投资计划，市儿童福利大楼、韶关市中心血站基本完成主体工程，帮助市区水上居民上岸定居正在实施。

八、政府自身建设加强，行政效率进一步提高

依法接受市人大及其常委会监督，执行人大决议，重大事项向人大常委会报告。依照章程接受市政协的民主监督，向政协通报工作情况，吸纳各民主党派、工商联、无党派人士和人民群众建议意见。组织实施市人大议案，办复市人大代表建议126件、市政协建议案4件、委员提案164件、委员来信63件，办复率100%，人大代表和政协委员普遍表示满意或基本满意。加强与各人民团体联系。颁发规范性文件13个，清理废止15个。取消、停止、免收行政事业性收费91项。推进本级政府机构改革。全面实行“政府信息、政务、事务、厂务、村（居）务”公开。深入开展“五五”普法。加强基层民主政治建设。完成47个社区办公用房建设任务。

认真落实党风廉政建设责任制和反腐倡廉工作部署。广泛开展“抓作风、塑形象”廉政教育系列活动，努力打造“诺廉、考廉、述廉、评廉”的廉政品牌，政府工作人员的廉洁自律意识进一步增强。加强重点领域、重点部门和重点项目资金使用的行政监察和审计监督，整治单位私设“小金库”，严查腐败案件。开展工程建设领域突出问题专项治理，深入治理商业贿赂，切实纠正损害群众利益的不正之风。加大公务员培训力度，公务员队伍整体素质提高。邓火坚被评为全国“人民满意的公务员”。深入开展机关效能监察、执法监察、政风行风评议、绩效考评以及机关作风暗访，机关服务质量和水平有新提高。解决群众通过行风热线投诉的问题1276个。简化行政审批和办事程序，入驻市行政服务中心的72项行政许可事项承诺时限比法定时限提前，平均提速达44%，行政审批提前办结率达99%，按时办结率为100%，人民群众对行政机关为民办事的满意率在90%以上。

过去一年，我市成功举办首届丹霞地貌国际学术讨论会，完成丹霞山申报世界自然遗产年度工作任务，联合国有关专家在考察评估中予以充分肯定。全国爱卫会命名我市为“国家卫生城市”，创建国家园林城市工作通过专家组实地考查。中国有色金属工业协会授予我市“中国锌都”称号。我市成功举办2009广东禅宗六祖文化节和2009广东国际旅游文化节韶关分会场系列活动，成功举办2009韶关（广州）招商引资推介会和2009韶关（东莞）招商引资推介会。在第二届中国城市盛典市长（香港）投资峰会上，我市夺得唯一的“2008—2009年度中国最具人气的旅游城市奖”。在去年12月新华社《经济参考报》、商务部中国国际经济技术交流中心和中国城市经济杂志社共同举办“2009中国经济发展论坛”上，我市被评为“2009中国经济科学发展十佳城市”之一。

各位代表：2009年是我市经济发展遇到巨大困难的一年，能够实现保增长、保民生、保稳定的目标十分不易。这是全市人民齐心合力、艰苦奋斗的结果。在此，我代表市人民政府，向全市广大工人、农民、知识分子和各级干部，向驻韶人民解放军、武警部队官兵和人民警察，向各民主党派、人民团体和各界人士，致以崇高的敬意！向长期关心支持韶关发展的港澳台同胞、海外侨胞、驻韶中省单位干部职工和国内外朋友，表示衷心的感谢！

在肯定成绩的同时，我们也面临不少的困难和问题。主要有：一是经济发展水平低，调结构、促发展的任务艰巨；二是国际金融危机影响依然存在，巩固工业和外经贸回升势头任务艰巨；三是财税减收因素多、增支压力大，保障社会建设和改善民生任务艰巨；四是区域、城乡发展差距仍然较大，一些群众生活困难状况亟待改善，救助救济和扶贫开发任务艰巨；五是一些部门重审批轻服务，加强政府自身建设和优化投资环境任务艰巨；六是各种不稳定因素仍然存在，一些信访突出问题有待进一步解决，维护全市社会稳定工作任务艰巨。对此，我们将高度重视，在今后工作中着力加以解决。

2010年工作安排

2010年是“十一五”规划的最后一年。做好今年工作，全面完成“十一五”规划目标任务、并为“十二五”发展打下良好基础，对实现韶关跨越发展，跟上全省现代化步伐意义重大。

去年以来，世界经济呈现恢复性增长。今年，我国继续实施积极的财政政策和适度宽松的货币政策，加快转变经济发展方式，着力保持经济平稳较快发展。我省加大提升珠三角、带动东西北工作力度，加快粤港澳经济一体化进程；狠抓促进粤北山区跨越发展的指导意见的落实，加大对山区发展的政策扶持；重点培育“五个一百”，加快“三旧”改造，实施扩大内需战略，推进基本公共服务均等化和主体功能区建设。我市随着武广高速铁路和韶赣高速公路相继建成通车，现代化交通枢纽功能日趋完善，投资环境将得到巨大提升。必须抢抓这些历史性发展机遇，进一步增强信心，鼓足干劲，全力促进增长、转变方式、增强后劲、普惠民生，努力实现经济社会又好又快发展，开创生态文明发展新局面。

2010年市政府工作的总体要求是：高举中国特色社会主义伟大旗帜，以邓小平理论和“三个代表”重要思想为指导，深入学习实践科学发展观，认真贯彻落实十七大和十七届四中全会、中央经济工作会议和省委十届六次全会、粤北地区（韶关）现场会、粤北山区工作会议精神，按照市委十届七次全会确定的发展目标任务，继续解放思想，坚持改革开放，全力以赴贯彻落实珠江三角洲地区改革发展规划纲要和省委、省政府关于实施扩大内需战略的决定、关于促进粤北山区跨越发展的指导意见，围绕做大经济总量这一首要任务，突出转变经济发展方式这一核心，进一步推动“三促进一保持”，抓好“两个关键”，重点打好转变经济发展方式、提升“双转移”工作质量、推进扶贫开发“规划到户、责任到人”工作这“三场硬仗”，加快产业和城市“双转型”，进一步壮大经济实力，注重加强环境保护和生态文明建设，全面建设名符其实的粤北区域中心城市，为“十二五”科学发展打下坚实基础。

2010年经济社会发展主要预期目标是：全市生产总值增长11%左右，全社会固定资产投资增长20%，完成省下达的节能减排任务，社会消费品零售总额增长15%，地方财政一般预算收入增长9%，实际利用外资保持增长，外贸出口增长8%，城镇登记失业率控制在3.5%以内，居民消费价格指数涨幅控制在3%左右，服务业增加值占生产总值比重达44%，全社会R&D投入占生产总值比重达1.6%，城镇居民人均可支配收入增长8%，农村居民人均纯收入增长8%，人口自然增长率控制在7‰以内。

今年要突出抓好以下八个方面的工作。

一、抢抓机遇扩大投资，全力夯实科学发展基础

抢抓实施扩大内需战略的机遇，创新项目工作方式和机制，加强项目策划、实施和储备，着力扩大投资规模，确保全年固定资产投资超过400亿元。加强与国家和省有关部门的沟通，重点落实珠江三角洲地区改革发展规划纲要对接项目和省关于促进粤北山区跨越发展的指导意见确定的项目，争取更多的政府投资项目和中央预算及省配套资金。完成好重点项目投资195亿元任务，确保乐昌峡、湾头水利枢纽和西气东输二线韶关段等在建重大基础设施项目顺利推进。继续推进韶关核电项目前期工作。力争韶关电厂“上大压小”项目开工建设，华电热电冷联供、广东国华太阳能光伏发电等能源项目加快推进。进一步优化投资环境，着力扩大民间投资和外来投资规模。

优化支出结构，增加民生支出，提高推进国企改革、全民创业、产业转移、旅游发展等专项资金使用绩效，有力带动企业和社会投资。加强财政支出管理，提高预算透明度。大力培育和扶持重点税源，狠抓税收征管，确保财政收入稳步增长。坚持银政企联席会议制度，完善信贷考核激励机制，强化莞韶、城投公司和政策性担保公司等融资平台功能，完善投融资体制，努力保持新增贷款较快增长。扩大信用担保规模。推进小额贷款公司等新型农村金融机构试点。抓好农信社产权制度和管理体制改革。加强资本市场工作，力争中小企业上市融资有实质性突破。

大力统筹城乡、区域协调发展，做大做强县域经济。加强对县域发展的规划指导、政策支持、资金投入和政绩考核工作，完善县域经济社会发展考核考评制度。支持县域城镇、园区的交通基础设施建设，加快城镇化步伐和特色经济发展。支持县域社会事业加快发展，促进城乡基本公共服务均等化。扎实推进富县强镇事权改革，转变县、镇政府职能，扩大县（市、区）管理权限。

二、坚持“工业强市”之路，全力加快新兴制造业基地建设

加大帮扶企业力度，推动工业稳步较快回升。对规模以上工业企业重点帮扶，促进增产增效、加快发展。加大力度培育发展有潜力的科技创新型企业。力争全年新增规模以上工业企业100家以上。加强工业经济运行分析监测，强化生产要素的协调，努力保障企业生产需要。深入实施工业“绿灯行动”，完善服务机制，加强对重点区域、重点产业、重点企业、重点项目的服务。坚持实行领导班子成员和部门联系服务重点工业企业、工业项目等制度，支持、帮助企业解决生产、经营、技改等方面的困难。

狠抓工业项目建设，着力培育新增长点。完善工业项目协调机制，加强全程跟踪落实和帮扶服务，力争一批大项目早日落地、加快建设、投产达效。重点推进韶钢节能减排、丹霞冶炼厂技改扩建、韶关比亚迪汽车试车场及零部件、华彩（翁源）化工涂料城、新丰中华制漆、乳源赛普超硬材料、东阳光生物制药、韶铸技改、大宝山铜硫采选、粤北危险废物处理处置中心等项目建设。力争工业固定资产投资上新水平。

转变工业发展方式，加快产业聚集发展。贯彻落实自主创新行动计划，深入实施知识产权战略。大力支持企业加大技术改造和科技研发投入，攻克产业发展关键技术。推广应用信息技术，加快工业化与信息化融合发展。实施企业科技特派员计划，加快成果转化，促进传统产业优化升级，力争有一批企业进入省“五个一百”行列。加强产学研合作，推动液压机械装备产学研创新联盟成为行业发展技术服务平台，推进中国科学院韶关产业技术创新与育成中心、中国科技开发院韶关高科技产业孵化基地、华南理工大学韶关技术研究院等重大公共技术服务平台共建工程，加快科技企业创业园（孵化器）建设与项目孵化，争取建成省级高新技术产业开发区。加大钢铁、有色金属、机械装备、玩具产业集群培育力度。争取申报建立省级铅锌及制品质量监督检验中心，加快有色金属材料、液压油缸和铝箔等特色产业基地建设。推进省市共建（韶关）装备基础零部件先进制造业基地，强化机械装备整机产品和关键零部件研发，整合机械装备工业资源，提升协作配套和整机生产能力。做强制药、电力、烟草产业，加快精细化工、电子与新型材料、稀有金属、固体废物处理等新兴产业发展，推进现代产业体系建设。

大力推动全民创业，加快民营经济和中小企业发展。全面落实民营经济和中小企业扶持政策，完善服务体系，加快推进市中小企业创业基地建设。努力破解民营经济和中小企业融资难问题，引导金融机构加大信贷支持力度。深入实施中小企业成长工程，鼓励中小企业发展“专、精、特、新”产品或技术。

三、继续掀起“两个高潮”，不断提升“双转移”工作质量

继续掀起园区基础设施建设高潮，加快提升产业承载能力。加强土地征用、报批和拆迁安置工作，重点保障园区发展用地需要。加大资金筹措力度，加快推进园区基础设施建设，完善园区配套服务。加快莞韶产业转移园打造省级示范性产业转移园步伐，有效推进各县（市、区）特色园区开发建设。

继续掀起招商引资高潮，全力以赴抓项目引进。进一步优化投资环境，完善招商服务体系。建立强有力的招商引资工作推进机制，力争引进一批大项目、好项目。大力实施《韶关市2009—2010年重点产业招商工作方案》，抓好钢铁及有色金属、机械装备及汽车零部件、电子信息、医药、农业及农产品加工业、物流、旅游、文化、体育等产业招商。做好已落户我市的大型企业的延伸招商工作，鼓励企业增资扩股。加强与东莞、深圳、广州等市的产业对接合作，积极承接产业转移。建立招商引资绩效评估和考核机制，提高项目工商注册率、资金到位率和开工投产率。加快转变外贸发展方式，努力促进出口稳定增长。加快实施市口岸通关物流中心项目，提升铁海联运和口岸通关服务水平。

加强人力资源培训配置，全面实施人才强市战略。加快出台人才激励机制，进一步优化吸引创新、创业领军人才等高层次人才的环境。鼓励和引导创新型人才、高校毕业生向企业流动。发挥市人力资源市场作用，加强农村劳动力技能培训转移就业工作。超额完成省下达的全年农村劳动力培训和转移就业计划。创新体制机制，整合优势资源，打响职教品牌，加快省市共建粤北现代技工职业教育基地步伐，努力把韶关建成全省最具特色的职业技术教育基地、全国一流的农民工培训示范基地。

四、落实和完善强农惠农政策，实现“三农”工作新突破

加强农田水利设施和农村建设。坚决打击非法占用耕地行为，严格保护耕地。加快现代标准农田建设，推进土壤改良和保护性耕作。完成城乡水利防灾减灾工程和病险水库除险加固工程建设任务。加快粤北现代农业示范园区建设。推进农业良种良法工程和农业科技进村入户工程，深化基层技术推广体系改革，完善农村科技服务网络。加快农村饮水安全工程建设。开展旧村庄改造和“空心村”整治，推进农村安居工程，完成泥砖房改造1万户以上。完成乡村公路硬化 400公里 。完善土地流转机制，推进农业集约化发展。

大力发展优质、高效、生态、安全的特色农业。着力调整优化农业结构，扩大特色种植业规模，提高畜牧水产品质量；突出发展经济林，提高林业产出，培育发展生态林业。抓好100万亩优质蔬菜、100万亩优质稻、300万头生猪和1亿只家禽、100万亩特色精品农业产业基地建设。加快农业龙头企业和农民专业合作组织发展，推进农业产业化。落实生态农业发展规划，推进有机农产品规划建设。落实粮食工作政府负责制。抓好集体林权制度改革，建立完善与现代林业发展相适应的管理服务体系，力争在今年内基本完成改革任务。加快发展良种油茶、珍贵乡土树种、竹子和林产品精深加工等特色林业产业。

扎实推进扶贫开发。全面推进“规划到户、责任到人”工作，坚持“一村一策、一户一法、整村推进”，落实帮扶责任，着力改善贫困地区发展环境，建立结对帮扶机制，加快脱贫步伐，力争以优异成绩通过省考核。开展村级农民互助金试点。落实对口帮扶责任，引导和鼓励社会各界参与扶贫开发，加快革命老区、少数民族地区、边远山区和水库移民区建设发展。

五、着力扩大消费需求，促进现代服务业持续繁荣

做大做强旅游产业。完善政府主导、市场主体的旅游机制，做好省国民旅游休闲计划试点城市工作，推进旅游强市建设。抓好丹霞山申遗工作，充分发挥申遗预期效应。加快“大丹霞、大南华、大南岭”等重点景区开发建设，推进丹霞山创建国家 5A 级景区和全国文明景区，建设环丹霞山旅游度假带，完善丹霞山旅游主通道，科学开发南岭森林公园。发展乡村旅游和自驾游，培育温泉产业，建设一批旅游休闲计划示范点和示范旅行社。强化城市旅游功能，建好游客服务中心，完善旅游服务体系，优化旅游消费环境。加强旅游营销和宣传，打响韶关生态休闲旅游品牌。

推进物流现代化。抢抓韶赣高速公路建成带来的机遇，逐步把我市建设成沟通泛珠三角的南北物流走廊。加强物流平台建设，推进韶关物流业对接广州和赣南、湘南。加快曲江、浈江、仁化等重点综合物流园区规划建设，支持优势物流企业发展壮大。大力推进电子商务、特色街区、专业市场、物流配送、连锁经营等现代流通业发展。

扩大居民消费。大力贯彻国家鼓励消费的政策，推进“万村千乡”市场工程和“家电下乡”、“汽车下乡”活动，继续开展家电、汽车“以旧换新”工作。引导商贸连锁经营向农村地区延伸，完善农村流通网络。支持城镇居民改善住房条件，促进房地产业健康发展。加强市场监测和信息引导。推动换代消费，开展信用消费，抓好节庆消费，促进消费需求持续快速增长。

六、加快芙蓉新城开发建设，打造粤北区域中心城市

加快推进芙蓉新城土地报批、拆迁安置、项目建设、多元融资、资产经营工作。启动韶关站周边地块开发。推进“三纵三横”路网及配套市政设施、公交站场建设。开工建设市区东环路。加快东堤路商业街改造。推进马坝组团全面对接融入中心城区。加快推进芙蓉新城至白土、韶关学院至大塘的公路建设，抓好韶冶至南华温泉路段改造和106国道南华寺段改道工程。加强城乡结合部、中心城市周边城镇的基础设施建设，完善交通安全设施。进一步解决农业人口在市区就业、落户等问题，提高城市化水平。

提高中心城市建设管理水平，巩固提升创卫创园成果。大力推进“三旧”改造，促进城市面貌更新，盘活城市存量建设用地资源。推进环卫、园林及市政设施维护等事业单位改革。加强城区和周边镇环卫基础设施建设。抓好各项绿化、美化、亮化工程。着力解决市区饮食店油烟污染、噪声扰民等问题。加快市区公交发展。加强“三江六岸”、城市公园、休闲广场、历史街区和绿道的规划建设，提升城市生态休闲功能和文化品位。积极推动进城农民有序逐步融入城镇，推动新农村建设与城镇化互动互促。

加快实施生态文明建设规划，着力打造全国生态文明建设示范市。加大节能减排和污染防治力度，确保完成省下达的任务。抓好跨界河流污染治理。加强生态环境保护和外来有害生物监测防控工作，高度重视生态经济、生态功能、生态人居以及生态文化建设。加强环境监管，确保生态安全。推行清洁生产，发展环保产业、低碳经济和循环经济，推进建筑节能。抓好灭荒造林、生态公益林林分改造、石漠化综合治理和“万村绿”工程。加强节约集约用地和矿产资源管理，进一步规范采矿秩序。全面完成污水处理厂建设任务。全面实施乡村“清洁美”工程，综合整治农村环境。加强村镇规划建设管理，抓好宜居城镇、宜居村庄建设试点，推进宜居城乡建设。

掀起新一轮交通建设高潮。加快赣韶铁路建设，确保韶赣高速公路按期建成通车、广乐高速公路韶关段全线动工建设。加快粤湘、昆汕、大广高速公路韶关段及韶关至贺州铁路项目前期工作。抓好国、省道改造和路面大修工程，重点抓好省道246线二期改建工程和省道347线回龙至牛仔峡改线工程。建成梅乐公路。建设芙蓉新城客运枢纽站和韶关南（曲江）客运站。加强韶关港新港港区、乌石港区和大坑口港区及韶钢、中海油等专用码头建设前期工作。争取北江航道韶关段按通千吨船等级标准纳入省内河航运发展规划。

七、全面发展社会事业，加大改善民生力度

开展创建全国文明城市工作。积极推进“六好”平安和谐社区、文明单位、文明窗口、文明村镇创建活动，引导和鼓励市民参与社会公益活动，大力提倡团结互助、扶贫济困的良好风尚，倡导文明健康的生活方式，全面促进社会文明进步。加强公民思想道德建设，弘扬爱国主义、民族精神和时代精神。加强国防建设、教育和动员，做好民兵预备役和人民防空工作，巩固“双拥”工作成果。

推进创建教育强市工作。加快义务教育规范化学校建设，加大农村中小学布局调整力度，加强城镇薄弱学校改造，推动城乡义务教育均衡发展。继续抓好普通高中和中等职业技术教育发展，巩固提高普及高中阶段教育成果。加强教师队伍建设和学校管理，大力实施素质教育，进一步提高教育质量。继续抓好学前教育、特殊教育、民办教育，支持高等院校提升办学水平，促进各类教育协调发展。

提升文化软实力。加大投入，加快文化事业发展。深化文化体制改革，创新文化事业单位体制机制。继续实施文化惠民工程，完善覆盖城乡的公共文化服务体系，促进群众文化繁荣发展，支持乳源承办好第十一届中国瑶族“盘王节”。完成省广播电视无线覆盖工程建设，加快市区有线数字电视整体转换。加强文化遗产保护利用，认真做好抗战时期中共广东省委暨粤北省委机关旧址的保护维修。继续做好第三次全国文物普查工作。做好城市语言文字规范工作。抓好文艺精品创作。加强文化市场管理，促进文化产业健康发展。

深化医药卫生体制改革，加强公共卫生、城乡基层医疗服务体系、县级医疗卫生机构标准化建设，进一步健全城乡医疗卫生服务网络。加强基层医疗卫生队伍建设。提高新型农村合作医疗保障和服务水平。深入实施中医药强市战略，推进区域医疗服务中心建设。推进实施国家基本药物制度，强化用药指导、监管和宣传，确保群众用药安全，减轻群众负担。巩固提高农村药品监督网和供应网，加强城市社区和农村食品药品监管。

继续完善和实施积极的就业政策，完善公共就业服务体系。建立健全公共投资促进就业增长机制。加大社区服务和公益性就业岗位开发力度，多渠道扩大就业。完善鼓励自主创业的扶持政策和服务体系。帮助就业困难群体、大中专毕业生就业。力争全年新增城镇就业5万人，失业人员再就业4万人。加快多层次养老保障体系建设，建立健全城乡居民社保体系，扩大社会保障覆盖面，提高保障水平，切实保障困难群体基本生活。支持工会做好困难职工帮扶工作。做好被征地农民养老保险、优抚对象和城镇居民医疗保障工作。完善重点优抚对象医疗保障制度。抓好新型农村社会养老保险试点扩面工作，争创“新农保”示范市。加强社会救助、救灾救济、法律援助和慈善工作。加强社会福利机构建设。逐步免除就读中等职业学校的农村经济困难家庭学生和涉农专业学生的学费。抓好天子岭、十里亭廉租住房和经济适用住房建设，加快东岗岭棚户区改造，扩大住房保障覆盖面。

加强维稳、安全生产、公安消防和森林防火工作。提高社会服务和管理水平，实行流动人口居住证制度。做好第六次全国人口普查工作。加强信访工作，推进镇街综治信访维稳中心建设，努力化解人民内部矛盾。健全防灾减灾体系，加强预警预测工作。完善突发事件应急体系，加强应急管理工作。加强市武警支队力量和装备建设，启动武警指挥中心建设。加强公安“三项”建设，抓好社会治安综合治理，适时开展专项行动，严厉打击犯罪行为，维护社会和谐稳定。

加强人口和计划生育工作，扎实开展全国人口和计划生育综合改革示范市创建活动，提高人口计生优质服务水平。推进体育事业发展。深入贯彻落实《全民健身条例》，承办好第四届广东省少数民族传统体育运动会和第十六届广东中华文化流动杯老年人门球赛，积极备战和组团参加广东省第十三届运动会。进一步做好民族宗教、外事侨务、对台、港澳、气象、水文、防震减灾、无线电管理、档案、审计、物价、统计、供销、史志、红十字会、老龄、妇女儿童、残疾人等各项工作。

八、切实加强自身建设，努力打造人民满意政府

提高依法行政水平。依法接受人大及其常委会的法律监督和工作监督。坚持重大事项向人大报告，积极主动向政协通报工作。认真接受政协的民主监督。认真听取社会各界、基层群众的意见和建议，接受媒体监督和社会监督。认真办理人大常委会的监督意见和人大议案、代表建议及政协建议案、委员提案。支持人大代表、政协委员履行职责。全面提升公务员队伍能力，打造一支作风优良、干事创业的干部队伍。规范行政执法行为，认真实施《行政复议法》、《行政许可法》，严格执法监督和执法责任追究。推进行政权力规范、公开、透明、高效运行，强化行政监督，提高行政效能，优化政务环境。坚持科学民主依法决策制度，科学编制我市“十二五”发展规划。

严格落实廉政建设责任制，广泛开展以“树立正确的权力观”为主题的廉政教育活动，构建“教育、制度、监督、改革、惩处、纠风”的惩治和预防腐败体系，筑牢有韶关特色的拒腐防变防线。完善廉政建设领导体制和工作机制，进一步落实“一岗双责”。强化制约监督机制，规范权力运行，重点加强对工程招投标、土地出让、环境保护、产权交易、政府采购、资源开发、项目建设等领域的监督。深入开展工程建设领域突出问题专项治理，推进“政府信息、政务、事务、厂务、村（居）务”公开。进一步加强机关效能监察，大力开展纠风专项治理，切实维护群众利益。严肃查处违纪违法和商业贿赂案件。稳步推进县级权力公开透明运行机制试点工作。

深化大部门体制改革，进一步精简机构、理顺关系、转变职能、提升效能。全面实施部门绩效考核和行政问责。深入开展民主评议政风行风活动。加强“民生热线”、“网络问政”和市长信箱工作，与人民群众保持密切联系，着力解决关系群众切身利益的热点、难点问题。今年办好十件实事：1、完善农村气象信息服务体系。2、实施天子岭廉租住房建设项目。3、实施乡村“清洁美”工程。4、更新50辆公交车及改造建设市区部分公交候车亭。5、启动异地兴建市特殊教育学校。6、实施林桥坑二期整治工程。7、建设市中心业余体校。8、改造市区中山公园文化广场中心舞台。9、建设粤北第三人民医院住院大楼。10、建设省消防战勤保障中心韶关基地。

各位代表：韶关加快发展的条件已经具备，我们肩负神圣的使命和历史的重任。让我们更加紧密团结在以胡锦涛同志为总书记的党中央周围，在省委、省政府和市委的坚强领导下，以更加强烈的责任感和紧迫感，抢抓机遇，勇于开拓，更新观念，跨越发展，圆满完成“十一五”规划，为建设粤北区域中心城市、争当全省山区科学发展排头兵而共同奋斗！
